# Impaired Object Handling during Bimanual Task Performance in Multiple Sclerosis

**DOI:** 10.1155/2014/450420

**Published:** 2014-08-06

**Authors:** Stacey L. Gorniak, Matthew Plow, Corey McDaniel, Jay L. Alberts

**Affiliations:** ^1^Department of Health and Human Performance, University of Houston, 3855 Holman Street, Garrison 104U, Houston, TX 77204, USA; ^2^Centers for Neuromotor and Biomechanics Research and Neuro-Engineering and Cognitive Science, University of Houston, Houston, TX 77204, USA; ^3^Frances Payne Bolton School of Nursing, Case Western Reserve University, 10900 Euclid Avenue, Cleveland, OH 44106-4904, USA; ^4^Cleveland FES Center, Louis Stokes VA Medical Center, Cleveland, OH 44106, USA

## Abstract

We investigated the kinetic features of manual dexterity and fine motor control during a task that resembles an activity of daily living in 30 persons with relapsing-remitting multiple sclerosis (PwMS). Specifically, a novel two-transducer system was used to measure time and grip-load forces during a bimanual task that is similar to opening and closing a jar. We hypothesized that PwMS would have increased grip force production, deteriorations in kinetic timing, and preserved grip-load coupling indices compared to healthy controls (i.e., young and older adults). Increased grip force production and deterioration in timing indices were confirmed in PwMS. Abnormal grip-load coupling was exhibited by PwMS, in contrast to healthy participants. The correlation between task time and self-reported disability scores suggests that objective measurement of impaired upper-extremity movements relates to perception of overall function.

## 1. Introduction

Many activities of daily living (ADLs), such as eating, bathing, and grooming require the hands to work in concert with one another to perform tasks using objects and tools. Appropriate coordination of applied fingertip forces is crucial for the successful performance of these activities. An inability to appropriately coordinate applied finger forces leads to consequences such as object slip, unintended object rotation, or damage to handheld items [1–3]. These unintended actions negatively impact the ability to perform daily activities that require fine motor skills. It has been widely shown that individuals with neurological diseases (e.g., multiple sclerosis (MS) and Parkinson's disease (PD)) exhibit motor deficits that produce abnormal fingertip force production during manual tasks [4–9].

Specifically, persons with MS (PwMS) exhibit abnormal grip force control in basic manual tasks [7, 8, 10]. PwMS exhibit significantly larger grip forces and altered modulation of grip-load force coupling during grasp of static objects [8, 10], as well as during simple lift and grip tasks [7, 8]. Evidence of deterioration in force control timing has been provided in dynamic tasks [7], with contradictory results in static tasks [8, 10, 11]. While these studies have provided insight into basic features of manual performance affected by MS, the consequences of altered manual function in MS have not been evaluated in realistic bimanual actions. Since PwMS commonly experience problems with upper-extremity tasks that require fine motor function [12, 13], it will be important to identify the particular kinetic deficits experienced in bimanual actions that resemble daily tasks. By identifying specific kinetic deficits during upper-extremity tasks, interventions can be more precisely tailored to target underlying impairments that improve upper-extremity function and the ability to carry out daily actions.

Recently, a two-transducer system has been developed that now enables evaluation of a more realistic and frequently performed bimanual task, that is, opening and closing of a jar [14] using different configurations of the hands to open the lid and stabilize the jar. Fundamental task-specific changes in bimanual behaviors have been observed between young healthy controls and older adults [14, 15]. Specifically, the particular task performed by the hands (e.g., use of rotation versus nonrotation) alters within- and between-hand force coordination patterns. Although altered grip force production exists in PwMS, it is unknown whether altered grip force production results in changed coordination patterns during realistic bimanual tasks. Furthermore, given the daily variability in fatigue and other MS symptoms, it is unknown whether force production and coordination patterns would be consistent during test-retest evaluations.

The purpose of the current study is to evaluate the force coordination patterns generated by PwMS during a bimanual task and examine whether these force coordination patterns are consistent in a two-week test-retest evaluation [7, 10]. We expected PwMS to produce larger grip forces and larger slip safety margins in bimanual tasks as compared to healthy young controls and healthy older adults (Hypothesis 1). In accordance with previous observations in PwMS, within-hand indices of force coordination exist in PwMS [8, 10]; however, we expected task-related differences in force coordination with object rotation and overall goals of the manual performance (Hypothesis 2). We also expected to see deterioration in force control timing mechanisms during dynamic actions of the hand (Hypothesis 3). Lastly, we expect that force coordination patterns will be consistent in test-retest evaluation (Hypothesis 4).

## 2. Methods

### 2.1. Participants

Thirty (30) adult females with a physician confirmed diagnosis of relapsing-remitting multiple sclerosis volunteered to participate in this study (48 ± 9 years old; mean ± SD); all PwMS were recruited from a randomized controlled pilot trial [16]. The average duration from time of diagnosis was 9 ± 7 years and the average duration from onset of symptoms was 14 ± 8 years. The majority of PwMS (28/30) were strongly right-handed according to their preferential use of the hand during daily activities such as writing, drawing, and eating. PwMS self-reported their perceived level of disability via the validated Patient Determined Disease Steps (PDDS) [17].  Table 1 contains PDDS scores for the tested sample. PwMS had no previous history of additional neuropathies, musculoskeletal disorders, severe cognitive deficits, or trauma to the upper limbs. All participants gave informed consent according to the procedures approved by the Institutional Review Board of the Cleveland Clinic. Test-retest sessions were two weeks apart. Data from PwMS in this study were compared to published normative data collected using the same device on two healthy populations: healthy young controls (*n* = 12; average age ± SD = 27 ± 6 years [14]) and healthy older adults (*n* = 10; average age ± SD = 66 ± 8 years [15]).

### 2.2. Experimental Setup and Procedure

**Figure 1:**
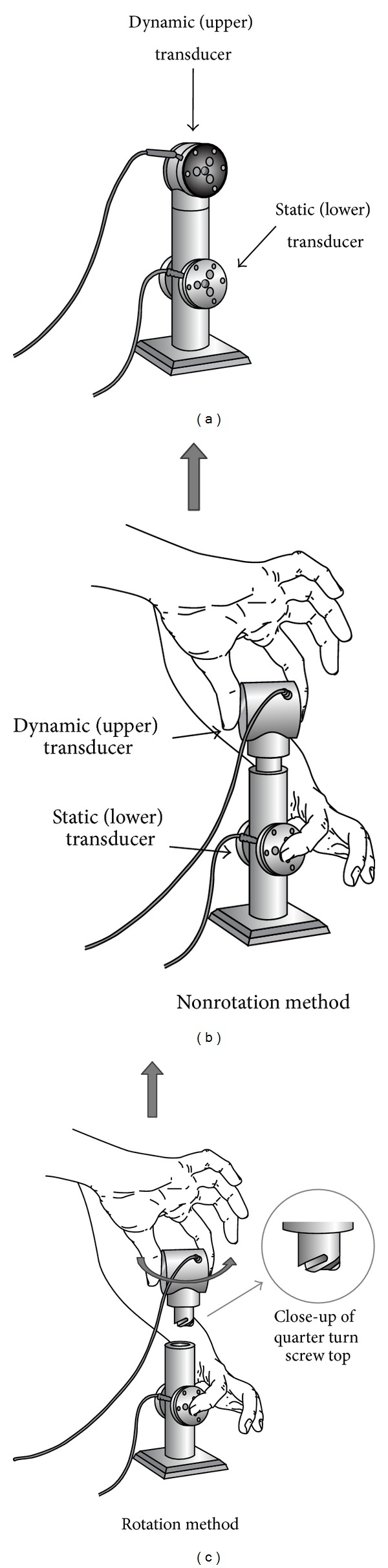
Schematics of the bimanual tasks examined in this study. (a) The device with sensors embedded in aluminum housings. (b) An example of the task performed with the nonrotation method; static and dynamic transducers are indicated. (c) An example of the task performed with the rotation method. A quarter turn rotation in the counter-clockwise direction was required when performing tasks with the rotation method, as shown in the magnified view of the articulation.

A system, as described in [14], was used to determine the time and force characteristics of commonly performed bimanual tasks. The task involved connecting two independent objects together using one of two movements: (a) placing one object on top of another (nonrotation) and (b) connecting the two objects by rotating the upper object while stabilizing the lower object (rotation). Grip and load forces of both hands were recorded simultaneously using two identical six-component force-moment transducers embedded in aluminum housing (Mini 40 transducers; ATI Industrial Automation, Garner, NC, USA). Two different articulation types (simple cylinder and a quarter turn screw top, referred to as* nonrotation* and* rotation *tasks, resp.) between the two objects were used (see Figures 1(a)–1(c)).

Participants were instructed to perform two different tasks for each articulation type, using thumb and index fingers only. Both the upper (dynamic) and the lower (static) objects were freely moveable; however, the location and orientation of the lower object were prescribed on the surface of a table at the onset of the experiment. In disconnect-type tasks, the dynamic object was initially attached to the top of the static object. In disconnect-type tasks, the dynamic object was placed in the upright position in a foam containment box located 20 cm in the horizontal direction towards the hand maneuvering the dynamic object. At the onset of connect-type trials, the dynamic object was located 20 cm horizontally away from the static object (toward the hand that would contact the dynamic object), positioned upright in a foam containment unit. In connect-type tasks, the dynamic object was attached to the top of the static object at the end of each trial. Subjects were not permitted to move the static object during testing.

Overall, four different bimanual configurations were tested. Five trials were collected in each of the four tested configurations (20 total trials in this experiment). Briefly, each bimanual task involved either the connecting of two separate objects into one object (connect) or disconnecting of an object into two separate objects (disconnect). This action was performed using the dominant hand to perform rotational (rotation) and nonrotational (nonrotation) actions of the upper/dynamic transducer. Subjects were instructed to begin each trial with both hands placed palm down on the surface of the table. The presentation of the four testing conditions was block randomized. The finger pad-transducer coefficient for static friction was assumed at 0.76 for the young control group and MS group [14, 15]. The finger pad-transducer coefficient for static friction was assumed at 0.42 for the older adult control group [15].

### 2.3. Data Analysis

Transducer signals were amplified and multiplexed using a customized conditioning box (from ATI Industrial Automation) prior to being routed to a 16-bit analog to digital converter (PCI-6036E, National Instruments, Austin, TX, USA). A customized LabVIEW program (National Instruments, Austin, TX, USA) was used for data acquisition and customized MATLAB (Mathworks Inc., Natick, MA, USA) programs were written for data processing. Signals were sampled at 256 Hz. The force data were low-pass filtered at 6 Hz using a second-order, zero-lag Butterworth filter [18, 19]. The use of 6 Hz as the cutoff frequency was verified via fast Fourier transform analysis. Force onset was defined as the earliest time of 3% maximal grip force application prior to the time point of actual maximal grip between the two transducers, within a trial. Force termination was defined as the time of 3% maximal grip force application after the time point of actual maximal grip of the dynamic transducer. Task time was defined as the period between force onset and force termination. All force data were time normalized with respect to task time (expressed as 0–100% of task time).

### 2.4. Temporal Analysis

Three measures of timing were calculated for this study. Task time, force onset delay, and time lag in grip-load force coordination (assessed via cross-correlation) were calculated for each subject in each condition. In the first two measures, averaged data were calculated across the individual trials after time normalization, aligned by grip force onset. For the cross-correlation time lag, values were determined from raw data. Positive lag values indicate that grip force changes lead load force changes; negative lag values indicate that load force changes lead grip force changes. All temporal measures are reported in seconds (s). Task time was defined as the time between grip force onset and termination. Force onset delays were defined as the differences in onset of forces applied to the two transducers; a positive value indicates that the static transducer was contacted initially while a negative value indicates that the dynamic transducer was contacted initially.

### 2.5. Kinetic Analysis

Grip and load forces were analyzed in terms of total load force and total grip force. The forces were calculated as twice the measured value of each force, respectively. This calculation was used as only two force-torque sensors were available for use in the experimental setup. Values of slip safety margin (SM, the amount of grip force exerted beyond what is required to prevent object slip) were computed using the traditional equation [20–22] SM = (*F* ^*G*^ − |*F* ^*L*^|/*μ*)/*F* ^*G*^, where *F* ^*G*^ is the grip force applied to the object, *F* ^*L*^ is the load-bearing force applied to the object, and *μ* is the coefficient of friction between the finger pad and transducer interface. Thus, the maximum value for SM is one if no load-bearing force is exerted on the object and the minimum value for SM is zero if just enough force is exerted on the object to prevent slipping.

Beyond general force computations, four correlation coefficients to assess force modulation were calculated (*rLoad, rGrip, rGL*_*D*_, and* rGL*_*S*_). Between-hands correlation for absolute load force (*rLoad*) was calculated as the correlation between the absolute value of total load force exerted on the dynamic transducer and the same value exerted on the static transducer. Similarly, the between-hands correlation for grip force (*rGrip*) was calculated as the correlation between the total grip force exerted on the two transducers. Within-hand grip-load correlation was calculated separately for the dynamic (*rGL*_*D*_) and static transducers (*rGL*_*S*_). These values were calculated as the correlation between the total exerted grip and load forces recorded by the respective transducer. Correlation coefficients were calculated as the overall correlation between the named variables across the entire time normalized interval (0–100%) in each of the tested conditions. Within-hand correlations (*rGL*_*D*_ and* rGL*_*S*_) were performed via cross-correlation, with the maximal correlation values being reported in the results. Correlation coefficient (*r*) values from the regression analyses were subjected to Fisher *z*-transformation to mitigate the ceiling effects inherent to these variables. Nontransformed data are presented in the figures to avoid confusion.

### 2.6. Statistics

The data are presented in the text and figures as means ± standard errors. Repeated measures analyses of variance (RM-ANOVAs) were performed on the force data with the between-subjects factor of* diagnosis *(three levels: MS, healthy young, and healthy older adult) and within-subjects factors of* method *(two levels: rotation and nonrotation) and* task *(two levels: connect and disconnect). An additional within-subjects factor of* CorrType *(four levels:* rLoad, rGrip*,* rGL*_*D*_, and* rGL*_*S*_) was used to compare the values of the correlation coefficients calculated in this study. Subscripts *D* and *S* refer to the within-hand correlation values for the dynamic and static transducers, respectively. To evaluate test-retest reliability, standard paired *t*-tests for pre- and posttest values were also performed.

To analyze differences between the measures recorded independently by the two transducers in the setup for the* rGL* measures, a within-subjects factor of* object *was used (two levels: dynamic and static). Further evaluation of differences in force coordination indices indicated by the CorrType results was evaluated for each index (*rLoad, rGrip*,* rGL*_*D*_, and* rGL*_*S*_) with separate RM-ANOVAs. Differences within levels of* diagnosis* and* CorrType* factors were confirmed via Bonferroni corrected post hocs. For all ANOVAs, the assumption of sphericity was verified using Mauchly's sphericity test. If sphericity was violated, the degrees of freedom were adjusted as necessary using Greenhouse-Geisser corrections. A covariate consisting of PDDS score was included in RM-ANOVA analyses to evaluate concurrent changes in measured behavior and perceived disability.

## 3. Results

### 3.1. Timing

**Figure 2:**
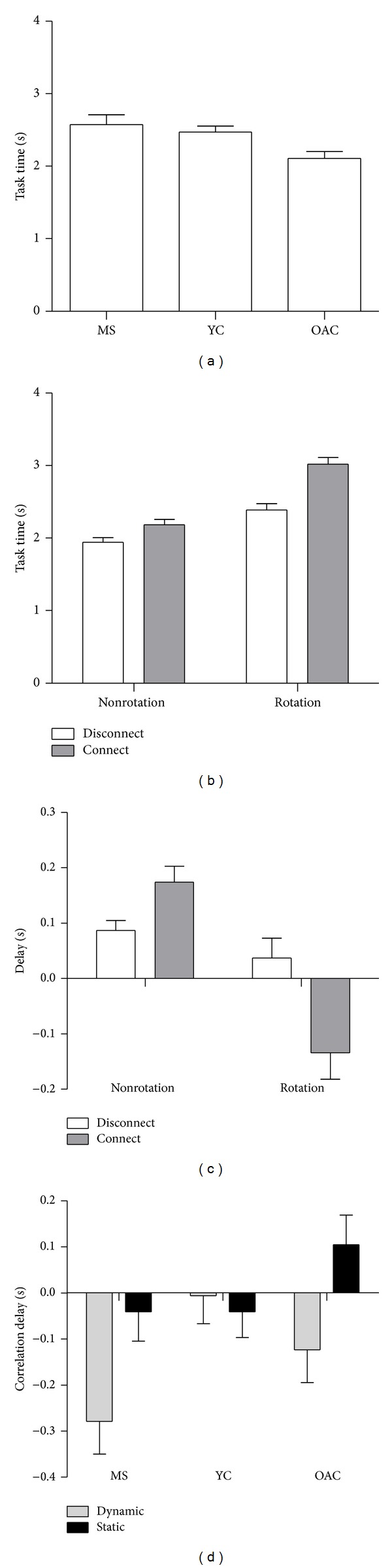
Mean and standard error of task time, force onset delays, and cross-correlation delays. MS: multiple sclerosis group, YC: young control group, and OAC: older adult control group. (a) Task time for each of the three diagnosis groups. (b) Task time across all three groups during rotational and nonrotational actions. (c) Force onset delays across all three diagnosis groups during rotational and nonrotational actions. (d) Cross-correlation delays for each of the three diagnosis groups.

The overall time to perform the task (task time) was slightly longer for PwMS than times exhibited by healthy participants (*diagnosis*:* F*_1.7,31.9_ = 6.4, *P* < 0.01). In particular, task times were longer for PwMS versus healthy older adults, verified via post hoc (Figure 2(a)). Task time in PwMS was positively covaried (*r* = 0.42) with PDDS score (*F*_1,19_ = 10.2, *P* < 0.005). Task time was affected by the* task *and* method* used. Task time was greater when the two objects were being connected to each other (*task*:* F*_1,19_ = 155.9, *P* < 0.001) and during rotational actions (*method*:* F*_1,19_ = 98.1, *P* < 0.001) shown in Figure 2(b). Task time increased when the two objects were being connected together; this effect was particularly larger when rotational actions were used (*task* ×* method*:* F*_1,19_ = 34.2, *P* < 0.001), also shown in Figure 2(b).

The difference in timing of force onset between the two transducers did not depend on* diagnosis*. Across all three groups, the force onset delay was negative when the two objects were being connected to each other, indicating that the dynamic hand performed the initial contact; however, the same delay was positive when the two objects were being disconnected from each other (*task*:* F*_1,38_ = 28.6, *P* < 0.001), indicating that the stabilizing hand contacted its respective transducer first during such tasks. Force onset delays were affected by the interaction of* task* and* method* (*task* ×* method*:* F*_1,38_ = 29.5, *P* < 0.001), such that trials in which objects were handled without rotation generally exhibited positive force onset delays, whereas connect-type tasks were negative, shown in Figure 2(c).

Analysis of cross-correlation time delays did not reveal task- nor method-specific differences in within-hand grip-load force coupling. In contrast to evaluation of healthy individuals, who exhibit 0 ms grip-load coupling time delays [14, 15], maximal grip-load correlation values for PwMS were found to consist of a near significant −160 ± 36 ms delay between the grip and load force profiles (*diagnosis*:* F*_2,57_ = 8.9, *P* = 0.064). Within-hand force coupling delays were larger for forces exerted on the dynamic transducer (−136 ± 39 ms) versus the static transducer (−8 ± 36 ms) (*transducer*:* F*_1,57_ = 8.2, *P* < 0.01). A* diagnosis* ×* transducer* interaction effect indicated that PwMS had significantly longer delays in within-hand force correlation, particularly for the dynamic transducer, as compared to healthy controls (Figure 2(d)) (*diagnosis* ×* transducer*:* F*_2,57_ = 3.4, *P* < 0.05). Note that negative lag values indicate that load force changes lead grip force changes.

### 3.2. Kinetics

**Figure 3:**
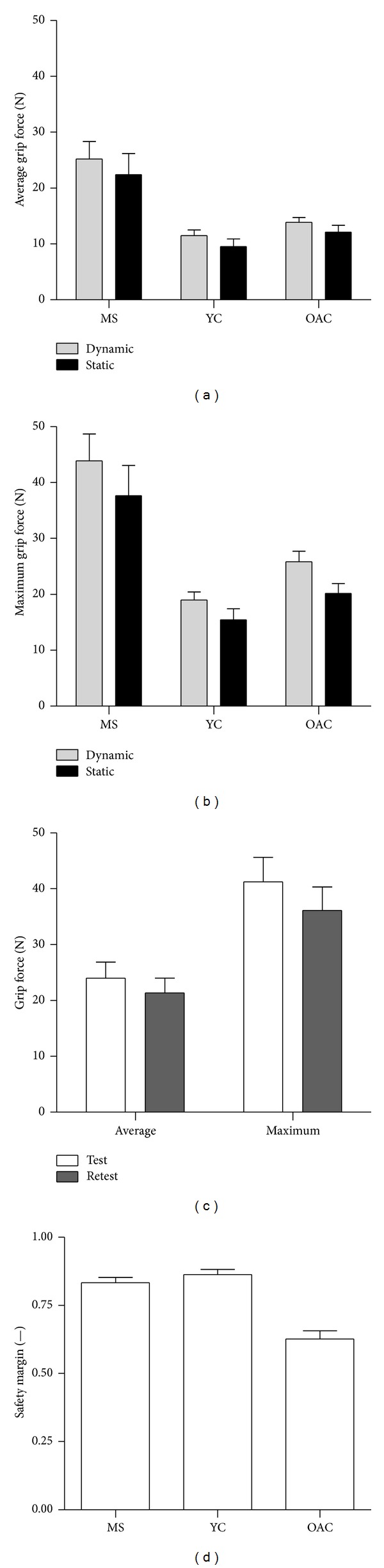
Mean and standard error of average grip force, maximum grip force, test-retest grip force values, and safety margins exerted. MS: multiple sclerosis group, YC: young control group, and OAC: older adult control group. (a) Average grip forces produced on the dynamic and static transducers. (b) Maximal grip forces produced on the dynamic and static transducers. (c) Grip force differences between test and retest sessions. (d) Safety margin values for each of the three diagnosis groups.

Compared to average (Ave) and maximal (Max) grip forces produced by healthy young and older adult individuals [14, 15], PwMS exhibited significantly larger grip forces in all tasks (Ave:* diagnosis*:* F*_1.1,20.7_ = 11.9, *P* < 0.005; Max:* diagnosis*:* F*_1.1,21.1_ = 15.5, *P* < 0.001). Excessive grip forces produced by PwMS were verified via post hoc. Across the three groups, higher grip forces were generally exerted on the dynamic transducer (Ave:* transducer*:* F*_1,19_ = 11.4, *P* < 0.005; Max:* transducer*:* F*_1,19_ = 37.3, *P* < 0.001), shown in Figures 3(a) and 3(b).

In contrast to all other test-retest measurements, exerted grip forces generally decreased between testing sessions for PwMS. Overall, grip forces were 11% lower in the retest session as compared to the test session (*P* < 0.05 for all *t*-tests, *R* for Ave grip force on dynamic transducer = 0.87, *R* for Ave grip force on static transducer = 0.88, *R* for Max grip force on dynamic transducer = 0.84, and *R* for Max grip force on static transducer = 0.88), shown in Figure 3(c). Despite this drop, grip forces produced by PwMS were still 1.5–2 times larger than those exhibited by healthy participants.

Safety margin (SM) did not differ among testing conditions across the three groups. PwMS exhibited similar SM values as compared to healthy young participants. PwMS and healthy young participants exhibited SM values larger than healthy older adults (Figure 3(d)). These results were verified via RM-ANOVA (*diagnosis*:* F*_1.32,14.5_ = 34.1, *P* < 0.001) and post hocs.

### 3.3. Correlations

**Figure 4:**
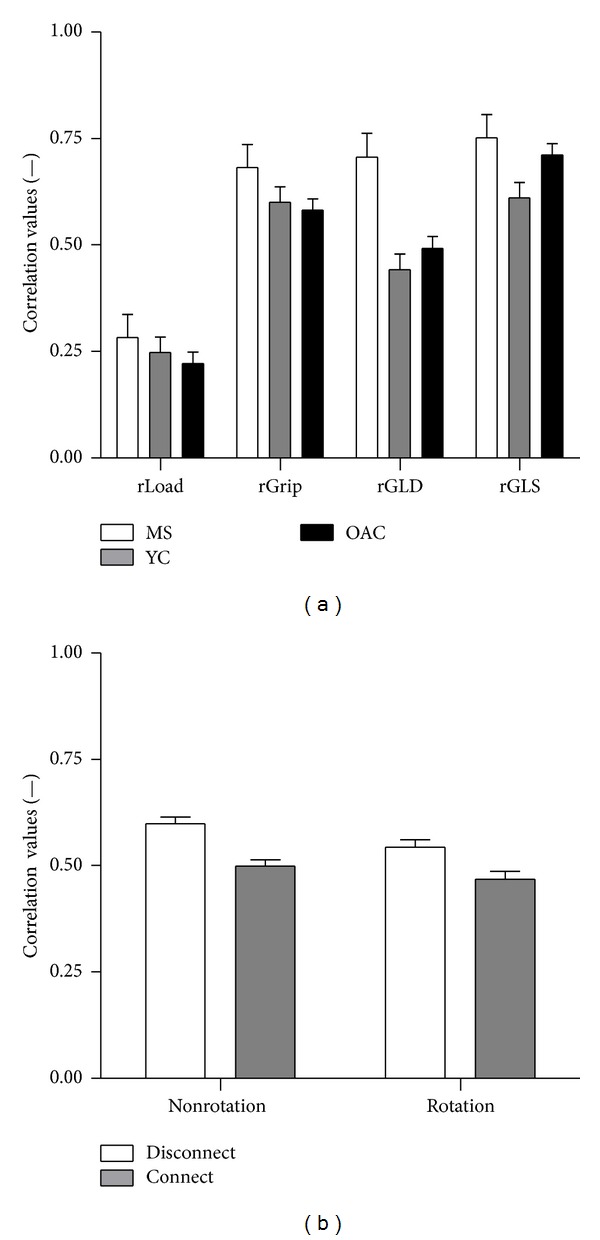
Mean and standard error of within-hand (*rGL*_*S*_ and* rGL*_*D*_) and between-hands (*rLoad* and* rGrip*) correlation coefficients by participants. (a) Correlation coefficients produced by each group. (b) Correlation coefficients in each task, averaged across diagnosis groups.

Within-hand (*rGL*_*D*_ and* rGL*_*S*_) and between-hand (*rGrip* and* rLoad*) force correlations were also evaluated. Force correlations exhibited by PwMS were higher than the values produced by healthy participants (*diagnosis*:* F*_1.5,109.3_ = 10.4, *P* < 0.001) confirmed via post hocs (Figure 4(a)) [14, 15]. Correlation values were lowest for between-hand load force coupling and highest for within-hand grip-load coupling exerted on the static transducer (*CorrType*:* F*_3,75_ = 82.4, *P* < 0.001), shown in Figure 4(a). The basic trend of weaker coupling in connect-type tasks (*task*:* F*_1,75_ = 33.8, *P* < 0.001) and during rotational actions (*method*:* F*_1,75_ = 7.0, *P* < 0.01) was consistent across groups (Figure 4(b)). Significant differences among correlation values were found, such that* rLoad* <* rGL*_*D*_ <* rGrip*,* rGL*_*S*_ was confirmed via post hocs (*P* < 0.013). All correlation values were found to be larger in PwMS compared to healthy participants [14, 15].

## 4. Discussion

Within the current study, we explored the effect of ecologically based bimanual actions on force coordination in PwMS. Consistent with previous studies, each of our four hypotheses was at least partially supported. Evidence of excessive grip forces (Hypothesis 1) with intact grip-load coupling changes based on task goals (Hypothesis 2) was exhibited by PwMS. Evidence of deterioration in grip-load force modulation and timing mechanisms was also exhibited by PwMS (Hypothesis 3). The majority of these features of bimanual performance were stable between testing sessions. Only grip force changed by 11% between test-retest sessions for PwMS yet remained 1.5–2 times higher than values produced by healthy participants, indicating that the current protocol is both reliable and robust in detecting functional differences in the majority of measures collected in this protocol (Hypothesis 4). In the following paragraphs, we discuss our findings in terms of the effects of MS on hand function.

### 4.1. Effects of MS on Hand Function

In previous investigations, PwMS have exhibited abnormal grip force control and altered modulation of grip-load force coupling during manipulation of handheld objects [7, 8, 10]. In the current data set, previous reports of overgripping in PwMS were confirmed with the bimanual task used in the current protocol with both static and dynamic components. The grip forces exerted by patients in this study were 1.5–2 times larger than grip forces exerted not only by healthy young adults [14], but also by healthy adults significantly older than the PwMS cohort given the same set of bimanual tasks with static and dynamic components [15]. As healthy aging has been associated with increased grip force production across a wide range of tasks [23–26], the statistically significant larger grip forces exerted by the PwMS group in this study suggest that overgrip in bimanual tasks with MS is not simply an effect of age; it appears to be a hallmark of the disease itself.

Despite task-dependent changes in the overgrip exerted by PwMS patients during manual tasks, the safety margin (the amount of grip force exerted beyond what is required to prevent object slip) was similar between healthy young controls and PwMS. In contrast, all within- and between-hand force coordination indices were larger in the PwMS group across all tasks, suggesting an inability to modulate finger forces due different task goals in MS. Even when accounting for the differences in cross-correlation delays in the PwMS group, the correlation among grip and load forces on the dynamic and static transducers (*rGL*_*D*_ and* rGL*_*S*_, resp.), correlation values were significantly higher in all conditions in the MS group compared to healthy participants [14, 15]. Additionally, values for between-hand force coordination (*rLoad* and* rGrip*) were also higher than healthy participants [14, 15]. This lack of force modulation, particularly in static and dynamic object manipulations [15], suggests significantly altered central control, possibility with respect to movement planning in PwMS [27].

Symptoms due to central deficits in MS are also supported by differences in timing found in the PwMS group [28]. Reports intact force timing coordination in PwMS has been contradictory in the literature [7, 8, 10, 11]. In the current study, time of task performance was significantly correlated with self-reported disability scores, suggesting that conscious perception of functional changes in MS does relate to objective measurement of impaired movement. In addition, PwMS showed significant timing differences between grip and load forces exerted on the handheld objects. Typically, zero-lag grip-load force cross-correlation delays are exhibited during manual tasks by healthy individuals [29, 30]. In contrast, the grip-load force cross-correlation delay exhibited by PwMS (cross-correlation delay = −160 ± 36 ms) indicates a loss in the ability to properly coordinate manual force production in the presence of the disease. Specifically, the negative lag value indicates that load force changes precede grip force changes in the PwMS group. Such discoordination of fingertip forces is likely to cause unintended object rotation during manual handling in PwMS [3]. This phenomenon suggests that the central nervous system (CNS) may not be able to fully couple the hands together as a functional unit in terms of timing and grip force control. Due to this deficit, PwMS may lose the ability to coordinate finer aspects of manual function while attempting to perform highly dexterous tasks such as buttoning a shirt or putting change in a parking meter.

Despite these findings, some evidence of task-specific changes in force production in PwMS was noted to be similar to the two healthy control groups in the current data set. This suggests that damage to the CNS in PwMS does not completely interrupt motor planning in this patient group. Instead, some task-specific characteristics are maintained, suggesting that the widespread scattering of MS lesions within the CNS may induce inflammation in multiple areas associated with motor function, thereby inducing some motor changes in PwMS [28, 31]. Last, the contribution of abnormal sensory function in MS cannot be ruled out and must be further investigated in future studies in this area. Given both the kinetic and timing differences exhibited by the PwMS group, kinetic markers may be useful as a preclinical marker of the disease.

### 4.2. Reliability of Protocol Used

In the current study, a protocol testing ecological bimanual function was evaluated in PwMS. This approach has been used to evaluate changes in bimanual hand function in other populations including healthy older adults [15] and individuals with Parkinson's' disease [5, 6]. In addition to the evaluation of bimanual hand function in PwMS, the design of the current study permitted test-retest evaluation of the bimanual testing protocol. Given the daily variability in fatigue and other MS symptoms, it was unknown whether bimanual coordination patterns would be consistent during test-retest evaluations in PwMS. Our results indicate that the current bimanual testing protocol is a reliable method for evaluation of hand function in PwMS. Of all measures collected and analyzed, only average and maximal grip force measurements differed between the test and retest sessions. All other measures remained consistent across testing sessions.

## 5. Conclusions

We conclude that MS is associated with increased grip force production, intact yet altered grip-load force coupling, and timing differences as compared to healthy young and older adults.

## Figures and Tables

**Table 1:** Patient determined disability status. Normal: some mild sensory symptoms. Mild disability: noticeable symptoms having a small effect on lifestyle. Moderate disability: no limitation on gait, but significant problems limiting ADLs in other ways. Gait disability: MS interferes with ADLs, particularly gait. Moderate cane use: can walk 25 ft/20 sec without assistance and always needs assistance to walk three blocks. Necessary cane use: assistance required to walk 25 feet.

Patient Determined Disease Steps (PDDS) score	Count
Normal	9/30
Mild disability	4/30
Moderate disability	2/30
Gait disability	5/30
Moderate cane use	5/30
Necessary cane use	5/30
